# Emerging biomarkers of immune checkpoint inhibitor toxicity in triple-negative breast cancer: a mini-review

**DOI:** 10.3389/fimmu.2026.1950429

**Published:** 2026-09-08

**Authors:** Erin McGillivray, Savannah Kane, Armina Saadatkhah, Kay T. Yeung

**Affiliations:** 1 Department of Internal Medicine, Moores Cancer Center, University of California, San Diego, La Jolla, CA, United States; 2 University of California, San Diego, School of Medicine, La Jolla, CA, United States

**Keywords:** biomarkers, ICI-related toxicity, immune checkpoint inhibitors, immune-related adverse events, triple-negative breast cancer

## Abstract

Immune checkpoint inhibitors (ICI) have revolutionized the treatment of TNBC, leading to durable responses and improved survival. With the adoption of immunotherapy into high-risk early-stage and selected metastatic triple-negative breast cancer (TNBC), clinicians’ ability to recognize and treat immune-related adverse events has become increasingly important. There is no prospectively validated or FDA-approved predictive biomarker of toxicity; however, candidate biomarkers involving circulating blood counts, immune-cell phenotypes, cytokines, transcriptomics, immune age, germline genetics, human leukocyte antigen genotypes, autoantibodies, and the gut microbiome have been described, with many more currently under investigation. Most evidence remains exploratory and is derived from non-TNBC populations. Going forward, incorporating these investigational tools within prospective TNBC trials and validating them in real-world, multicenter TNBC cohorts are critical to ensure these predictive biomarkers translate into clinically useful tools for risk-adapted monitoring. This mini-review highlights the current landscape of predictive biomarkers of ICI toxicity, with a focus on TNBC.

## Introduction

Immune checkpoint inhibitors have improved outcomes in early-stage and metastatic triple-negative breast cancer (TNBC) (1–3) but can lead to immune overactivation—known as immune-related adverse events (irAEs)—that can affect almost every organ system. These irAEs can cause treatment interruption, hospitalization, and irreversible organ dysfunction and can significantly impact patients’ quality of life. The optimal preventative strategy, balanced with maximizing tumor response, remains unclear as observational studies have reported associations between irAEs and improved cancer outcomes (4, 5). Recent breast cancer-specific studies suggest that this relationship may vary according to toxicity severity and treatment setting: mild-to-moderate irAEs were associated with improved outcomes in metastatic breast cancer, whereas severe irAEs were associated with worse survival (6). In early-stage TNBC, retrospective studies have reported variable associations between irAEs and pathologic complete response (7). No prospectively validated biomarker currently identifies individuals at sufficiently high risk of irAEs to guide the selection or omission of standard immunotherapy. Herein, we summarize emerging biomarkers for baseline risk stratification and longitudinal detection of ICI toxicities, with emphasis on TNBC-specific evidence, limitations, and priorities for clinical translation. Future studies should prospectively evaluate organ-specific and clinically actionable models embedded within TNBC trials and multicenter real-world cohorts.

### Pathophysiology of irAEs

Immune-related adverse events (irAEs) arise from a multifactorial breakdown of peripheral immune tolerance following checkpoint inhibition (8). While the exact mechanisms of irAEs remain incompletely understood, current evidence suggests that ICIs disrupt T-cell tolerance, increasing recognition of self-antigen and promoting autoimmune injury to normal tissues (9). ICIs may allow self-reactive T cells to escape cell death and undergo clonal expansion, while cross-reactivity between tumor and normal-tissue antigens may further contribute to organ-specific toxicity. Regulatory T-cell abundance or function may also be impaired, shifting the immune balance toward inflammation. Direct T-cell–mediated tissue injury, together with inflammatory cytokines, B-cell activation, and autoantibodies, likely contributes to the broad spectrum of irAEs (9).

### Clinical assessment, timing, and management of irAEs

IrAEs are graded using the Common Terminology Criteria for Adverse Events (CTCAE), which standardizes the assessment of adverse event severity (10). Decisions regarding ICI continuation, interruption, immunosuppressive therapy, and rechallenge are guided by organ-specific clinical practice guidelines and clinical judgment. The 2026 NCCN Guidelines for Management of Immunotherapy-Related Toxicities provide detailed recommendations for the evaluation and treatment of individual toxicities (11).

The median onset of all irAEs is approximately 40 days, with 15%–20% of patients experiencing a serious adverse event (12, 13). However, variations between cancer type and ICI class, as well as challenges with accurate reporting, make it difficult to determine the real-world timing and prevalence of these toxicities. Cutaneous toxicities generally occur early, whereas endocrinopathies and some pulmonary toxicities may develop later or even after treatment completion (14).

The toxicity profile most relevant to breast cancer is driven primarily by PD-1/PD-L1 blockade rather than CTLA-4-based therapy. Specifically, in early-stage TNBC, KEYNOTE-522 reported that 35% of patients experienced an irAE of any grade, while 13% had an irAE of grade >3 (15). The most common irAEs reported were hypothyroidism (15.1%), skin reaction (5.7%), hyperthyroidism (5.2%), gastritis (3.4%), adrenal insufficiency (2.6%), pneumonitis (2.2%), and thyroiditis (2.0%) (15). A 2026 systematic review and meta-analysis of ICIs in early-stage breast cancer similarly identified endocrine irAEs, particularly thyroid dysfunction, as among the most frequent immune-mediated toxicities, while severe irAEs were less common (16). Real-world studies have similarly demonstrated clinically meaningful rates of endocrine, cutaneous, pulmonary, hepatic, and gastrointestinal toxicities, although the reported incidence and association with pathologic complete response vary across cohorts (17–19).

Treatment of irAEs depends on the affected organ and toxicity severity. Many grade 1 irAEs permit continued therapy with close monitoring, whereas clinically significant grade 2 events often require temporary treatment interruption and administration of oral corticosteroids. Grade 3–4 irAEs usually require systemic corticosteroids or other immunosuppressive treatment, specialist evaluation, and suspension of ICI therapy. Some cases, such as endocrinopathies controlled with hormone replacement, may warrant treatment resumption; however, severe cardiac, neurologic, pulmonary, or recurrent toxicities may necessitate permanent discontinuation (14).

## Candidate biomarkers of ICI toxicity

**Figure 1:**
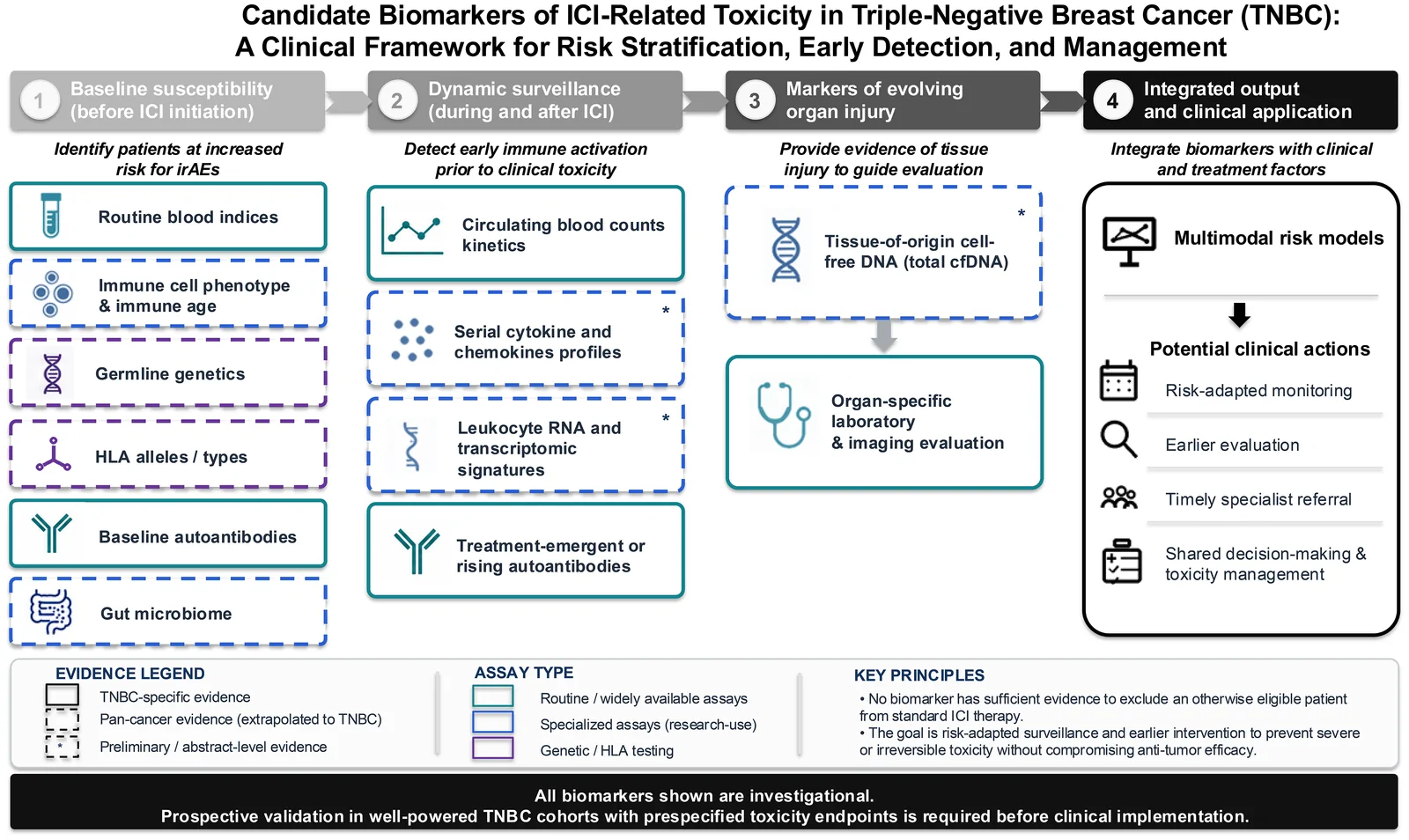
Emerging biomarkers of irAEs.

Candidate biomarkers may serve different clinical purposes. Pretreatment biomarkers estimate baseline susceptibility, while longitudinal biomarkers identify evolving toxicity during therapy, and diagnostic biomarkers help distinguish irAEs from infection, chemotherapy toxicity, disease progression, or other causes of organ injury. Because irAEs are biologically heterogeneous, a single biomarker is unlikely to predict all toxicities (**Figure 1**). Multimodal and organ-specific approaches may therefore be more clinically informative.

### Routine peripheral blood markers

Peripheral blood counts are routinely obtained in oncology practice and represent inexpensive potential biomarkers of irAEs. The most well-studied of these markers is the neutrophil-to-lymphocyte ratio (NLR), which is significantly elevated at the onset of irAEs (20). Kengkla et al. demonstrated that a baseline NLR ≥2.75 independently predicted any irAEs (adjusted OR, 2.44), was predictive of grade ≥3 irAEs (adjusted OR, 3.05), and was also associated with an earlier onset of irAEs (21). Multiple studies have reported that patients with higher absolute lymphocyte count (ALC) and absolute eosinophil count (AEC) are at increased risk of ICI toxicity (22–24). In another study evaluating lymphocyte fluctuations throughout treatment, patients who developed severe irAEs had an approximately 30% decrease in ALC at the onset of irAEs, suggesting that a decline in ALC during treatment could also predict toxicity (25). In TNBC, chemotherapy, growth-factor administration, infection, corticosteroid exposure, and treatment-related marrow suppression may substantially affect blood-cell indices. Routine blood counts are therefore unlikely to function as stand-alone biomarkers but rather as low-cost components of multivariable risk models.

### Circulating immune-cell phenotypes

High-dimensional immune profiling may provide a more mechanistic assessment of host susceptibility to irAEs. Using mass cytometry by time of flight (CyTOF) to profile leukocyte markers from pretreatment peripheral blood, Lozano et al. identified that higher baseline levels of activated CD4+ effector memory T (TEM) cells were significantly associated with severe irAEs in melanoma patients receiving ICI (26).

In patients with advanced breast cancer receiving ICI, higher baseline proportions of CXCR3+CCR6+CD4+ T cells and CD38+CD86+CXCR3+CCR6+CD8+ T cells, together with lower proportions of CXCR3lowCD56dim natural killer (NK) cells, were associated with irAEs (27). Similar patterns were observed in immune-related pneumonitis and thyroiditis subgroups. A prediction model achieved an area under the receiver operating characteristic curve (AUROC) of 0.79 in the training cohort and 0.75 in a single-cell validation cohort. This study is important because it is breast cancer-specific and biologically coherent; however, broader external validation, assay standardization, and confirmation in TNBC are needed before clinical implementation.

### Cytokines, transcriptomic signatures, and immune age

Cytokine profiling is among the most actively studied approaches to irAE prediction. Existing studies suggest that irAE risk reflects both baseline immune activity and treatment-induced change. Elevated baseline IL-17 has been associated with immune-related colitis, while lower baseline levels of CXCL9, CXCL10, CXCL11, and CCL19 have been reported among patients who developed irAEs (28, 29).

In patients with early-stage TNBC receiving pembrolizumab-based therapy, six markers (TNF-α, IL-10, IL-8, IL-12, ICAM1, and CXCL12) involved in NF-κB-related inflammatory signaling were identified in patients who developed irAEs (30). Low baseline levels of TNF-α, IL-10, and IL-8 were associated with the development of grade ≥2 irAEs. In contrast, rising IL-12p70 levels and declining MCP-1 levels were observed at multiple time points in patients receiving treatment who developed irAEs. In the GeparNuevo study, patients with TNBC who developed irAEs following durvalumab plus chemotherapy had higher pretreatment IFNγ, IL-7, and GM-CSF levels compared to posttreatment (31). Based on this hypothesis-generating evidence, multiple cytokine risk scores are under development (32–34). Of note, a composite serum cytokine score showed only a modest association with irAE risk (HR 1.4), underscoring the need for disease-specific calibration (30). However, small sample sizes, multiple comparisons, and heterogeneous assays limit interpretation, though they remain hypothesis-generating. These studies support serial immune monitoring rather than reliance on a single baseline measurement.

Finally, a novel SABCS 2025 Poster Spotlight study evaluated an “immune age” framework, characterizing patients’ intrinsic immune-system function and their risk of irAEs. Immune age was derived from 96 circulating inflammatory proteins using a trained linear regression model to predict chronological age. Immune-age acceleration was defined as the difference between immune age and chronological age. Thus, a positive immune-age acceleration indicates an immune phenotype that appears biologically older than expected based on chronological age and may capture baseline inflammatory status more directly than chronological age alone. Among 33 patients with early-stage TNBC treated with perioperative pembrolizumab and chemotherapy, 13 developed grade 3–4 irAEs. Higher baseline immune-age acceleration was associated with severe irAEs, whereas chronological age was not (35). This host-centric approach may help explain why age alone inconsistently predicts toxicity. However, the small TNBC cohort, limited number of events, and absence of independent validation preclude clinical use. Prospective validation of this assay and its threshold in a larger and diverse early-stage TNBC cohort is needed.

### Germline genetics, HLA, and autoantibodies

Germline biomarkers are appealing because they can be measured before treatment and are not altered by therapy. Genome-wide association studies have identified variants near IL7, IL22RA1, 4p15, and other loci associated with multiple irAE subtypes across multiple cohorts. The IL7 risk allele was associated with increased B-cell IL7 expression, altered immunoglobulin expression, B-cell receptor repertoires, and distinct treatment-induced CD8-positive T-cell responses (36, 37). However, most evidence derives from melanoma or pan-cancer populations, and none have been validated in TNBC.

Selected human leukocyte antigen (HLA) alleles and serologically defined HLA types have been associated with organ-specific irAEs. HLA-DRB1*11:01 was associated with pruritus (OR = 4.53), while HLA-DQB1*03:01 was associated with colitis (OR = 3.94) (38). In a Japanese cohort, HLA-DR15, HLA-B52, and HLA-Cw12 were enriched among patients who developed ICI-associated pituitary irAEs with secondary adrenal insufficiency. HLA-DR4 has also been associated with ICI-induced insulin-dependent diabetes (39, 40).

Autoantibodies have long served as markers of inflammation in autoimmune disease and are also thought to contribute to the development of irAEs. General autoantibody positivity has been reported in patients with ICI-induced skin toxicity, myositis, and endocrinopathies, with autoantibodies detected in almost 50% of patients with ICI-associated endocrinopathies (41). However, broad autoantibody positivity may reflect underlying immune dysregulation and is unlikely to predict a specific toxicity.

Organ-specific autoantibodies may be more clinically informative. ICI thyroiditis occurs more frequently in patients with pre-existing antithyroid antibodies, suggesting that baseline antibody positivity may identify increased susceptibility (42). Treatment-emergent antibodies or rising titers may instead indicate evolving immune-mediated injury. In one study of patients with solid tumors receiving pembrolizumab, a ≥1.5-fold increase in anti-thyroglobulin or antithyroid peroxidase antibody titers between baseline and the third treatment cycle was associated with irAEs (43).

More specifically, the phase II TREND study showed that 14 of 50 patients with early-stage TNBC receiving neoadjuvant tislelizumab plus chemotherapy developed thyroid irAEs. Baseline thyroglobulin antibody positivity (OR = 9.31) and lower free triiodothyronine levels were independent predictors. Higher T-cell and natural-killer-cell measures, tumor mutational burden, and exploratory KLF17 and LRIG1 mutations were also reported (44). Thus, baseline organ-specific autoantibodies may help predict susceptibility, whereas newly detected antibodies or rising titers may support early recognition of an evolving irAE. However, autoantibodies alone do not confirm an irAE and should be interpreted alongside symptoms, biochemical abnormalities, and organ-specific diagnostic evaluation. Prospective studies should determine whether baseline or serial autoantibody testing improves early diagnosis or reduces irreversible endocrine and neuromuscular toxicity.

### Gut microbiome

The gut microbiome has emerged as another tumor-extrinsic host factor that may influence both ICI efficacy and susceptibility to irAEs (45, 46). Across cancer types, differences in microbial composition and diversity have been associated with ICI outcomes, although reproducible toxicity-specific signatures remain poorly defined (46–48). Gut dysbiosis, rather than a single organism, has been implicated in irAE risk, as evidenced by a higher relative abundance of pathobionts and lower abundance of *Ruminococcaceae* in one prospective pan-cancer cohort of patients who developed severe irAEs (49). Similarly, antibiotic exposure, which can alter microbiome diversity, has also been associated with irAE risk, with one study showing that antibiotic use within 3 months before ICI or 1 month after treatment was a significant independent predictor of irAEs (P <0.001, OR: 6.152) (50). Various strategies to manipulate the gut microbiome have been explored, including prebiotics, fecal microbiota transplantation, and IL-6 blockade (51–53), although limited data exist in the TNBC space. Identifying patients who may be at high risk of severe irAEs based on their pretreatment microbiota could be clinically useful, but larger, prospective studies in patients with TNBC are warranted to identify reproducible toxicity-associated signatures and determine whether microbiome-informed risk stratification adds clinically meaningful information.

### Emerging biomarkers and multimodal approaches

While circulating tumor DNA can offer a minimally invasive window into cancer status and recurrence, total or tissue-of-origin cell-free DNA (cfDNA) has been recently proposed to monitor therapy-related toxicities. cfDNA released from damaged noncancerous cells rises rapidly in the blood and can be analyzed for tissue-of-origin-specific epigenetic markers, allowing for early detection of tissue toxicity before symptoms or conventional laboratory abnormalities (54). In the non-metastatic TNBC space, Huebner et al. used liquid biopsy to identify RNA expression from circulating leukocytes at different timepoints during treatment and found that CDK2, F5, and HLA-DR mRNA levels were associated with irAEs (55). A non-canonical TNFR/NF-κB pathway signature was inversely associated with irAEs in the durvalumab arm, and several baseline immune signatures correlated with tumor mutational burden. These findings provide proof-of-concept that peripheral immune signatures may be predictive of treatment response, survival, and toxicity in patients with TNBC receiving immunotherapy and chemotherapy. Further studies are needed to establish analytical sensitivity, specificity for immune-mediated injury, lead time, and clinical actionability.

Finally, multimodal machine-learning approaches may improve prediction by integrating complementary features rather than relying on a single biomarker. Bobe et al. developed a model combining T-cell and B-cell receptor repertoire embeddings with peripheral blood RNA-seq signatures to predict severe irAEs (56). The model was trained and evaluated across six cohorts including pre-ICI cancer patients, healthy donors, and rheumatoid arthritis (RA) patients. The combined TCR/BCR embedding plus gene signature model achieved an AUC of 0.78 for severe irAE prediction and 0.70 for RA detection, outperforming models based on gene signatures or TCR/BCR diversity alone. Although promising, predictive performance alone does not establish clinical utility. Future models should undergo independent validation and report calibration and performance across demographic and clinical subgroups. They should also be compared with simpler clinical or laboratory-based models and be evaluated prospectively to determine whether biomarker-guided monitoring or intervention reduces severe toxicity without compromising cancer outcomes.

## Conclusion

Immunotherapy has changed the treatment landscape of historically difficult-to-treat cancers like TNBC. However, navigating immune-related adverse events that often lead to treatment interruption, permanent organ dysfunction, and dramatic effects on quality of life remains a major challenge. No available biomarker has sufficient evidence to justify withholding immunotherapy solely due to predicted toxicity risk.

The near-term role of predictive biomarkers is more likely to involve risk-adapted surveillance, earlier diagnostic evaluation, and minimization of life-threatening, immune-related events. While there are multiple encouraging studies exploring relevant biomarkers to predict irAEs (**Figure 1**), all require validation in adequately powered prospective and independent cohorts to determine whether they are scalable and clinically relevant. Future studies should prioritize organ-specific, multimodal, externally validated, and actionable models embedded within prospective TNBC trials and real-world survivorship programs. Beyond predicting who will develop an irAE, the goal should be centered on preserving effective immunotherapy treatment while reducing irreversible harm to patients.
